# Optimal Chemotherapy for Leukemia: A Model-Based Strategy for Individualized Treatment

**DOI:** 10.1371/journal.pone.0109623

**Published:** 2014-10-13

**Authors:** Devaraj Jayachandran, Ann E. Rundell, Robert E. Hannemann, Terry A. Vik, Doraiswami Ramkrishna

**Affiliations:** 1 School of Chemical Engineering, Purdue University, West Lafayette, Indiana, United States of America; 2 Weldon School of Biomedical Engineering, Purdue University, West Lafayette, Indiana, United States of America; 3 Riley Hospital for Children, Indianapolis, Indiana, United States of America; University of Catania, Italy

## Abstract

Acute Lymphoblastic Leukemia, commonly known as ALL, is a predominant form of cancer during childhood. With the advent of modern healthcare support, the 5-year survival rate has been impressive in the recent past. However, long-term ALL survivors embattle several treatment-related medical and socio-economic complications due to excessive and inordinate chemotherapy doses received during treatment. In this work, we present a model-based approach to personalize 6-Mercaptopurine (6-MP) treatment for childhood ALL with a provision for incorporating the pharmacogenomic variations among patients. Semi-mechanistic mathematical models were developed and validated for i) 6-MP metabolism, ii) red blood cell mean corpuscular volume (MCV) dynamics, a surrogate marker for treatment efficacy, and iii) leukopenia, a major side-effect. With the constraint of getting limited data from clinics, a global sensitivity analysis based model reduction technique was employed to reduce the parameter space arising from semi-mechanistic models. The reduced, sensitive parameters were used to individualize the average patient model to a specific patient so as to minimize the model uncertainty. Models fit the data well and mimic diverse behavior observed among patients with minimum parameters. The model was validated with real patient data obtained from literature and Riley Hospital for Children in Indianapolis. Patient models were used to optimize the dose for an individual patient through nonlinear model predictive control. The implementation of our approach in clinical practice is realizable with routinely measured complete blood counts (CBC) and a few additional metabolite measurements. The proposed approach promises to achieve model-based individualized treatment to a specific patient, as opposed to a standard-dose-for-all, and to prescribe an optimal dose for a desired outcome with minimum side-effects.

## Introduction

### 1.1. Background on ALL & Maintenance Therapy

Acute Lymphoblastic Leukemia (ALL) is a malignant state of the bone marrow characterized by an abnormal and uncontrolled proliferation of lymphoblasts. In the U.S., yearly incidence of ALL is more than 5,000 [1] and is the leading class of cancer among children. Treatment for ALL comprises three phases. Newly diagnosed patients undergo remission-induction treatment using a combination of chemotherapeutics to bring down the tumor burden to less than 1% of the initial leukemic cells [2]. This is followed by consolidation therapy using high-dose of drugs to target specific locations of usual leukemic cell migration. The final phase of the treatment is maintenance therapy (MT) which utilizes relatively mild doses of drug over a period of 2–3 years to eradicate residual leukemic cells. Common acute side-effects during ALL treatment include but are not limited to myelosuppression, gastro-intestinal intolerance, hepatotoxicity, pancreatitis and neuropathy.

Having accomplished a high 5-year event-free-survival (EFS) rate of ∼85%, the next obvious focus is on minimizing acute and chronic treatment-related side-effects. This may include: i) identifying patient subgroups that are non-responsive and/or are prone to excessive side-effects and ii) choosing optimal dosage for each subgroup/patient. Though the 5-year EFS is obviously significant, and deceptively so in some sense, two thirds of the long-term survivors embattle treatment related late effects [3]–[5]. Recurrent ALL, secondary neoplasm and other multiple chronic medical conditions are prevalent among the survivors. They also encounter psychosocial issues, impaired quality of life and enjoy lower rate of socioeconomic advantages [3]. Clinical studies show that the administration of high dose of chemotherapeutic agents poses a significant risk towards long-term survival and quality-of-life among childhood cancer survivors [3], [6]–[9].

### 1.2. Importance and Issues of Maintenance Therapy

Maintenance therapy forms an important and indispensable part of the overall ALL treatment program. About 40% of the patients have minimal residual disease (MRD) at the end of consolidation; MRD is highly correlated with relapse rate [10]. Hence, a carefully designed and administered MT protocol is important to eradicate the residual disease. Patient self-administered oral combination of 6-Mercaptopurine (6-MP) and Methotrexate (MTX) is shown to improve the overall treatment outcome [10]–[12]. Clinical studies show that inadequate maintenance therapy leads to recurrent ALL whereas aggressive treatment results in acute side-effects and secondary malignancies, thus calling for the optimization and individualization of MT [13]–[17]. Hence, it is very important to monitor and optimize the treatment intensity during MT.

There are, among others, two major challenges encountered during MT of childhood ALL. First is the genetic polymorphism exhibited in the enzyme activity of Thiopurine Methyl-transferase (TPMT), an enzyme that plays a major role in conversion of 6-MP into various metabolites [18]. TPMT genetic polymorphism is directly correlated with treatment outcome and hence it is adopted as a companion diagnostic tool in many 6-MP protocols [14], [19], [20]. 6-MP is a pro-drug which undergoes intracellular metabolism involving two competing metabolic pathways [21]. The desired pathway leading to 6-thioguanine nucleotide (6-TGN) is catalyzed by an enzyme HGPRT. The catabolic pathways are catalyzed by TPMT which leads to the formation of various methyl-mercaptopurines (MeMP). The relative activities of HGPRT and TPMT are genetically transcribed and regulated for a given patient and dictates the net concentration of the active metabolite 6-TGN and hence the treatment outcome. For instance, in patients with high TPMT activity, 6-TGN pathway is suppressed, resulting in low 6-TGN concentration and hence treatment failure. On the other hand, in patients with low TPMT activity, encounter life-threatening myelotoxicity and treatment interruptions. Hence, it is prudent to utilize the TPMT genotype/phenotype as a basis to guide the treatment protocol for an individual patient [22].

Second is the inability to evaluate the treatment progression during MT due to the residual nature of the disease. Measurement of residual leukemic cells during MT remains elusive given the milder treatment condition and low turn-over rate of leukemia cells. During MT, bone marrow cell populations are exposed to 6-MP; when the cells move to the periphery, they retain the metabolized products. Thus, the concentration of 6-TGN in peripheral red blood cells (RBCs) is shown to indicate the systemic exposure to the chemotherapy agent and hence correlated to the treatment efficacy and toxicity [23]. Clinical studies show that the mean corpuscular volume (MCV) of the peripheral RBCs increases significantly due to the exposure to 6-TGN and provides an opportunity to be utilized as a surrogate marker for treatment monitoring [23]–[25]. Currently, in ALL MT, patients are initially treated with doses based on body surface area and are titrated to target neutrophil range of 1000–2000/µL. However, it is not possible to precisely judge the nadir of neutrophil level.

### 1.3. Importance of Individualized Treatment

Though mathematical models and optimal control theory have been utilized in almost all fields of science and engineering for over a century with tremendous contributions, they are not utilized to their fullest potential in medical applications [26]. Application of Mathematical approaches have shown excellent utilities in understanding and solving several issues in HIV, diabetes [27], [28]. Several aspects of cancer dynamics have also been studied through mathematical models [29]–[31]. Mathematical models, suitably empowered by systems theoretic methodology, derive their strength from their potential to quantitatively evaluate known or conjectured mechanisms of physiological processes. Clinical studies on childhood ALL show that some treatment regimens perform better than others [17], [32], [33]. A Pediatric Oncology Group study concluded that compared to twice-daily regimen, daily dosing regimen resulted in significantly higher metabolites of 6-MP [32]. In a recent study on Brazilian childhood ALL patients during MT [33], significantly higher 5-year EFS and overall survival rates were observed in intermittent treatment group. These results suggest that since one dose regimen performs better than the other, there exists an optimal regimen that performs better than many other regimens. Unfortunately, it is not possible to explore all possible combinations of regimens in a clinical trial.

**Figure 1:**
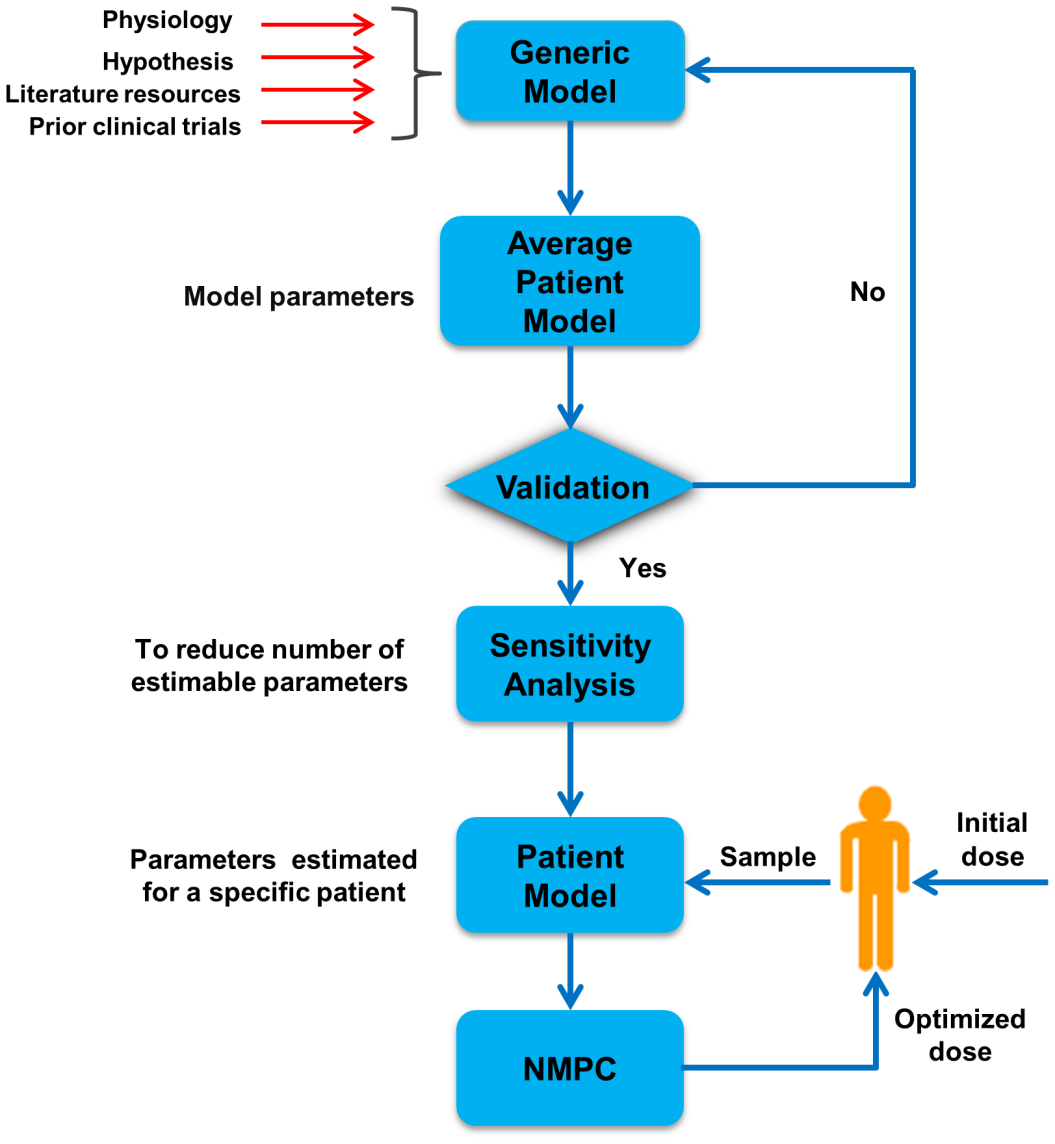
A generalized approach for model-based prediction and optimization of treatment outcome. A model representing the underlying physiology is formulated and average patient parameters are estimated using data from literature and past clinical trials. GSA is used to reduce the parameter space and identify most sensitive parameters with sparse clinical data. Following nominal initial dose, few measurements are taken from an individual patient in order to adapt the model to the patient. Patient-specific model is used to predict the treatment outcome and subsequent doses are optimized based on NMPC prediction.

We recently developed a semi-mechanistic model for indirect measurement of treatment efficacy using MCV of the RBCs as a surrogate marker [25]. The model considered a given concentration of active metabolite 6-TGN in RBCs and predicts dynamic changes of MCV in response to 6-TGN. Metabolism of 6-MP into 6-TGN and side-effects were not considered in that work. The present study aims to extend the model by explicitly incorporating the metabolism of 6-MP to 6-TGN and the major side-effect (leukopenia) during treatment. In addition, a simple and effective model-reduction technique based on global sensitivity analysis (GSA) is introduced to reduce the parameter space. This is important because personalization requires model adaptation to an individual patient through effective identification of parameters with sparse clinical data. The proposed modeling and sensitivity based approach will take into account the pharmacogenetic variation, through TPMT enzyme activity, to estimate the initial dose based on established therapeutic window. Once measurements (6-TGN, MCV and Leukocyte levels) are available following initial dose, patient specific sensitive parameters are identified. Once individual patient model is available, we used nonlinear model predictive control (NMPC) to optimize the dosage subject to a minimum level of WBC. The uncertainty in the patient-specific parameters associated with the treatment response models can be minimized by adaptively changing them according to the frequently available CBC data. The model is simple enough to be implemented in the clinical settings and requires few measurements of metabolites concentration and routinely measured CBC data to be able to adapt to individual patients. Although not modeled explicitly, implementation of such approach may help to improve the long-term EFS and quality-of-life among childhood ALL patients as they are the resultants of inappropriate dosing. The overall strategy is depicted in Fig.1. In section 2, we detail the methodologies utilized in this work including models to describe 6-MP metabolism, leukopoiesis and MCV dynamics. In section 3, we show some of the important results of this work with the simulation of virtual patients' response to 6-MP treatment. In sections 4, we discuss the results and implications of this work.

## Models and Methods

Let a generic dynamic model for any given drug and patient be given by a system of ordinary differential equations with the following form,
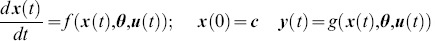
(1)where 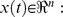
state variables, 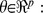
model parameters, 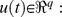
drug input, 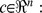
initial conditions, 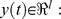
model output. In most circumstances 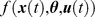 will be a nonlinear function and a numerical solution is readily available. For the purpose of this study, the parameter space 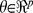 is divided into redundant parameters, 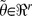 and sensitive parameters 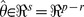. Sensitive parameters are identified for each patient whereas redundant parameters are fixed for all patients at average value.

### 2.1. Modeling 6-MP Metabolism

**Figure 2:**
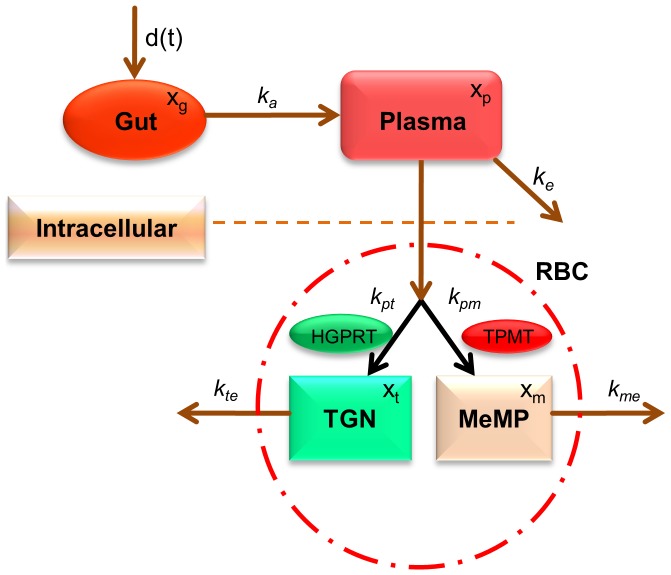
Schematic representation of 6-MP metabolism. Following oral intake to the gut, 6-MP is absorbed into the plasma from where it is eliminated through various routes. From plasma, 6-MP diffuses into the cells and enzymatically converted to 6-TGN and MeMP, which in turn are eliminated from the cells at a constant rate.

Although the TPMT pharmacogenomics and the metabolism of 6-MP is one of the extensively studied systems in clinical pharmacology literature, mathematical models are limited. We are only aware of the works of Hawwa et al. [34] where TPMT genotype was considered as one of the covariates to describe the inter-individual variations. However, many studies show that TPMT phenotype is a better marker than the TPMT genotype because there is a huge variation of TPMT enzyme activity within a specific genotype. A simplified schematic of the 6-MP metabolism accounting for two major metabolites is shown in Fig. 2. Following oral intake to the gut, 6-MP is absorbed at the rate of 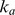 into the plasma where it undergoes first-pass elimination at the rate of 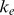. From plasma, 6-MP gets into RBCs followed by intracellular metabolism. There is a negligible concentration of 6-MP reported inside RBCs [35]; hence, we assume that 6-MP gets metabolized as soon as it enters RBC. 6-MP undergoes metabolism through two major pathways driven by HGPRT and TPMT leading to 6-TGN (active) and MeMP (inactive), respectively. Though there are several forms of MeMP produced, for modeling purpose, they are lumped into a single component collectively catalyzed by TPMT. 6-TGN and MeMP are eliminated from RBCs at the rates of 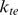 and 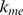 respectively. 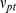 and 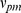 are included for unit consistency across equations. 
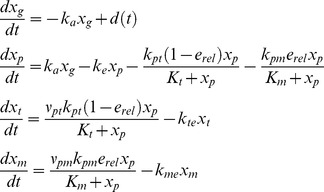
(2)


The model equations for 6-MP metabolism based on mass-action kinetics are shown in Eqn. 2. The description and units of all the state variables and parameters are listed in Table 1. The conversion of 6-MP into 6-TGN and MeMP is modeled using Michaelis–Menten (M-M) kinetics. Patient specific TPMT enzyme activity is represented as a relative activity level to their maximum level. Thus,
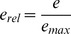
(3)where 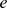 denotes TPMT enzyme activity. It is observed in the clinical studies that the production of 6-TGN is not limited by the HGPRT activity but is negatively correlated with TPMT activity [36]. Hence, it is assumed that a fraction 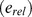 of 6-MP is converted to methylated metabolites based on the enzyme activity observed in a specific patient and the rest 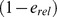 is converted to active 6-TGN.

**Table 1:** Glossary of state variables and parameters for 6-MP model.

Model variables	Description	Units
*x_g_*	Amount of 6-MP in Gut	pmol
*x_p_*	Amount of 6-MP in Plasma	pmol
*x_t_*	Concentration of 6-TGN in RBCs	pmol/8×10^8^ RBCs
*x_m_*	Concentration of MeMP in RBCs	pmol/8×10^8^ RBCs

Parameters	Description	Units	Values
*k_a_*	6-MP Absorption Rate from Gut	per day	4.8
*k_e_*	6-MP Elimination Rate from Plasma	per day	5.0
*k_pt_*	6-MP to 6-TGN Conversion Rate	pmol 6-MP converted per day	29.8
*K_t_*	MM Constant for 6-TGN	pmol	4.04×10^5^
*k_pm_*	6-MP to MeMP Conversion Rate	pmol 6-MP converted per day	655.8
*K_m_*	MM Constant for MeMP	pmol	3.28×10^5^
*k_te_*	6-TGN Elimination Rate from RBCs	per day	0.0714
*k_me_*	MeMP Elimination Rate from RBCs	per day	0.06
*e*	Actual TPMT Activity	Units per ml RBC	13
*e^max^*	Maximum TPMT Activity	Units per ml RBC	26
*ν_pt_*	Stoichiometric coefficient for 6-TGN Conversion	pmol 6-TGN produced per pmol 6-MP/8×10^8^ RBCs	1
*ν_pm_*	Stoichiometric coefficient for MeMP Conversion	pmol MeMP produced per pmol 6-MP/8×10^8^ RBCs	1

### 2.2. Modeling Treatment Response

In humans, blood cells (RBCs, WBCs, platelets etc.) are one of the most dynamic and vital population of cells with high turnover rates. Since most of the cytotoxic drugs, such as 6-MP, are designed to target fast-renewing cells, they invariably destroy bone marrow cells. Consequently, monitoring of peripheral blood count has become an integral part during chemotherapy to assess the bone marrow toxicity. However, this is a reactive rather than a proactive approach as the bulk of the damage is done to the immature, bone marrow cells instead of the peripheral cells. Since the maturation takes about 5–12 days, there will be a marked delay in the realization of the effects in the periphery. Thus, the prediction of the cellular dynamics during 6-MP treatment would help to forecast the oncoming decline in the cell population and adopt corrective measures before the patient contracts the side-effect. Mathematical models are in use to describe many facets of the hematopoiesis processes [37], [38]. Although mechanistic models are rich in physiology and are able to display diverse system behavior, they come with the price of several parameters. With the constraint on data, semi-mechanistic models of specific behavior within the exhaustive overall process would be of much use.

#### 2.2.1. Modeling Leukopoiesis

**Figure 3:**
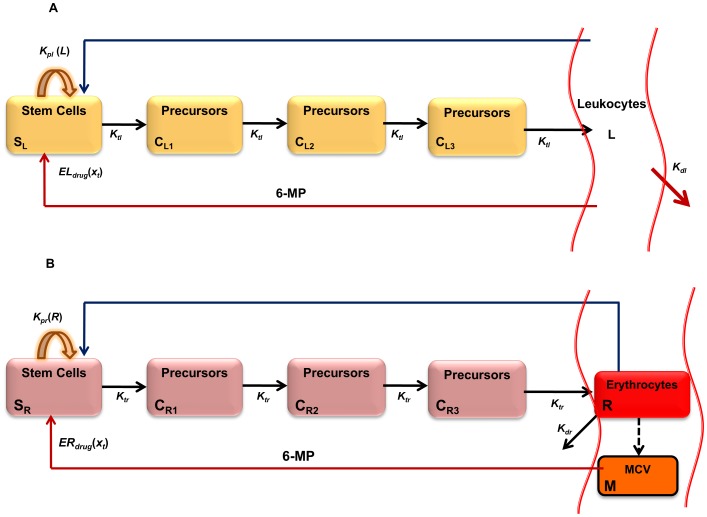
Simplified schematics of the leukopoiesis and erythropoiesis model. Stem cells reside in the bone marrow, proliferate, mature and enter the circulation as fully functional leukocytes. Stem cells receive biochemical feedback for proliferation from the circulating blood. On treatment initiation, 6-MP enters the bone marrow and imparts cytotoxicity to the stem cells. Leukocytes and RBC MCV in the circulating blood are routinely measured and used as a dose-limiting parameter. A. Leukopoiesis, B. Erythropoiesis. Additional compartment for MCV was added to account for the dynamic changes following 6-MP treatment. Solid arrows represent cellular movement; dashed arrow represents property changes.

The model to study the effect of chemotherapy on leukocytes was inspired by [39]. The schematic diagram of the leukopoiesis is depicted in Fig. 3A. The stem cells and early proliferating progenitors are depicted as compartment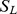. During early stages of stem cell differentiation, cells have the potential to proliferate. As they mature, they expel their nucleus and loose their proliferating potential [40]. 6-MP's cytotoxicity is due to incorporation of 6-TGN as false nucleotides into the DNA. Hence, 6-MP will have no influence on cells in the later stages lacking nuclei. Consequently, it is assumed that the cells in this compartment are the sole casualties of the cytotoxic drugs. Proliferation rate of the cells in this compartment is a function of leukocytes in the circulating system i.e. 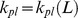. This feedback mechanism is mediated by a cytokine known as granulocyte–colony stimulating factor (G-CSF). The next three compartments, viz. 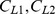
and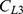, cater for the maturation process. Cells in these compartments are not susceptible to cytotoxic drugs as they are not proliferating. The fully matured, functional leukocytes enter the circulation, perform their functions for a specified period and die at a constant rate. 
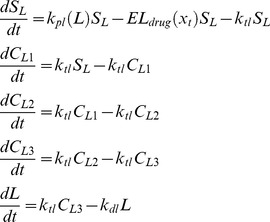
(4)


The equations for leukopoiesis model are given in Eqn. 4. The description and units for all the state variables and parameters for the leukopoiesis model are listed in Table 2. In the stem cell compartment, positive feedback for proliferation with amplitude modulated negatively by 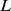 is represented using the following structure:
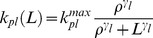
(5)


**Table 2:** Glossary of state variables and parameters for leukopoiesis model.

Model variables	Description	Units
*S_L_*	No. of proliferating progenitors	cells per kg
*C_L1_*	No. of maturing leukocyte precursors	cells per kg
*C_L2_*	No. of maturing leukocyte precursors	cells per kg
*C_L2_*	No. of maturing leukocyte precursors	cells per kg
*L*	No. of circulating leukocytes	cells per liter blood

Although the feedback is mediated by biochemical messenger G-CSF, the concentration of G-CSF is inversely proportional to circulating leukocytes as G-CSF is mainly cleared by the receptors on the leukocytes [41]. Unlike the term used in [39], which is a monotonically increasing function of leukocytes, Eqn. 5 has a saturating nature which derives its basis from receptor theory [42]–[44]. 6-MP effect in this compartment assumes the following Hill type kinetics. 
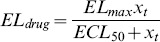
(6)where 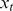 is the 6-TGN concentration obtained from 6-MP model. The cells are transferred to the maturing compartments at the rate of 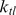. The matured cells enter the circulation at the rate of 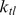 and die at the rate of 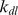.

#### 2.2.2. Modeling MCV Dynamics

Erythropoiesis, similar to leukopoiesis, is a process through which the body generates erythrocytes and hence assumes a similar model structure. The schematic diagram of erythropoiesis and MCV dynamics is shown in Fig. 3B. The proliferating fraction of the erythroid stem cells and early progenitors are represented as 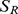. Cells in this compartment are assumed to be vulnerable to 6-MP. Proliferation rate of these cells is regulated by the number of RBCs 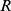. This feedback mechanism is mediated by a cytokine known as erythropoietin (EPO). 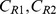 and 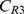 represent the maturing erythroid precursors. Fully functional RBCs enter the periphery, circulate for ∼120 days and die at a constant rate. MCV, which is the total volume of RBCs divided by the number of RBCs, in compartment 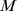 increases due to two phenomena: 1) inflow of new cells from the bone marrow (RBCs are larger when they enter the circulation and loose about 15% of the volume during their lifetime), 2) due to the administration of 6-MP. MCV decreases due to the death of RBCs which depends on the current MCV. When 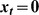, the only influence on MCV would be the imbalance in RBC production. At steady state (and without 6-MP), the two terms on the right hand side will cancel each other. 
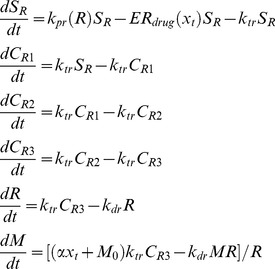
(7)


The model equations for erythropoiesis and MCV dynamics are given in Eqn.7. The feedback for proliferation amplification in stem cell compartment, was approximated by the inverse proportion of the circulating RBCs as follows,
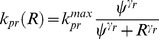
(8)


The effect of 6-MP treatment on this cell population is modeled using Hill type kinetics. 
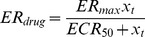
(9)


A linear relationship, with coefficient 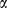, was assumed for the effect of drug on the MCV [23]. The loss of MCV was attributed to the death of the RBCs. The resulting total volume was normalized by the total number of RBCs. ΔMCV is calculated as the change in MCV from the baseline (untreated MCV) when 6-MP is administered. The description and units for all the state variables and parameters for the MCV model are listed in Table 3.

**Table 3:** Glossary of state variables and parameters for MCV model.

Model variables	Description	Units
*S_R_*	No. of proliferating erythrocyte progenitors	cells per kg
*C_R1_*	No. of maturing erythroid precursors	cells per kg
*C_R2_*	No. of maturing erythroid precursors	cells per kg
*C_R3_*	No. of maturing erythroid precursors	cells per kg
*R*	No. of circulating erythrocytes	cells per kg
*M*	MCV of circulating erythrocytes	femto liter (fL)

### 2.3 Parameter Estimation

Model parameters were estimated through minimization of sum of squared errors between model predicted values and the experimental data with the following cost function
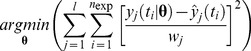
(10)where 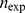 represents the number of experimental data points, 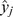 is the experimental data. When more than one variable is measured, appropriate weight, 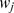 is used to normalize the cost function. Hybrid genetic algorithm is used as an optimization routine to perform the error minimization step and implemented using Matlab functions ‘ga’ and ‘fmincon’ [45]. Parameter bounds for optimization algorithm were chosen from feasible physiological ranges available in the literature.

### 2.4 Model Reduction through Global Sensitivity Analysis

Due to a large inter-patient variability in treatment response, the models developed here have to be adapted to individual patients' characteristics through parameters. Since clinical data are generally sparse, it is not possible to uniquely identify all the parameters as the system is underdetermined. This quandary can be addressed through model reduction using sensitivity analysis (SA) to identify redundant parameters in the model [46]. SA aims to distribute, either quantitatively or qualitatively, the variation in the model output to variation in the model inputs, e.g. parameters. Although it is reasonable to assume that the changes in the model input affect the model output, not all inputs influence the model identically. Thus, SA sheds light on the important model parameters that drive the model outputs. Since the model parameters vary widely among patients, we employ global sensitivity analysis (GSA). Soboí technique was used to estimate the total sensitivity indices [47]. Lower and upper bounds were chosen at 50% and 200% of the mean respectively. 1000 sets of parameter were sampled through sparse-grid technique. Although redundant parameters have little influence over the variable of our interest, they impart indirect effect through other auxiliary variables. Hence, redundant parameters are fixed at a nominal value (equal to that for an average patient) instead of being eliminated. Since any uncertainty in the estimation of the highly sensitive parameters will influence the model prediction greatly, it is prudent to estimate the most sensitive parameters as accurately as possible with the limited data available and be less concerned about the least sensitive ones. The error involved in such an approximation can be estimated as follows [47].

Let the set of parameters be 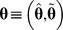 in which 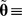 
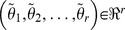
 represents the 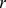 redundant parameters and 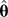 comprises the sensitive parameters. Further let 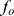 represent
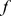
with 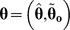 where 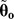 denotes the redundant parameters fixed at nominal value. For any redundant parameter 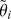, if 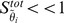, then the error of approximating 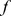 with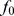, 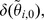 by fixing 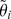 at a nominal value 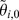 can be estimated by 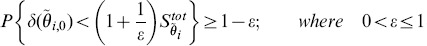
(11)where 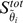 is the total sensitivity index of the model output corresponding to parameter 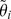. For an arbitrary value of 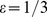, the probability of getting 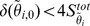 is more than 2/3.

### 2.5 Optimal Treatment Planning through NMPC

The final step in model-based individualized dosing is the determination of optimal dosage using patient-specific models. In this work, we utilize NMPC to optimize the dose due to its inherent nature of calculating the input based on the predicted system behavior subject to the state and input constraints as well as the optimization of a given cost function [48], [49]. In general, based on the measurement obtained at time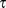, the controller predicts the evolution of treatment response over a prediction horizon 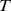 and estimates the dosing profile that optimizes the predetermined clinical objective function. To account for disturbances and model-patient mismatch, the optimization problem is solved for *finite* horizon but only the first dosing action is prescribed. The remaining samples are discarded and a new optimization problem is solved based on 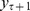 at the next clinical visit 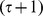. For the case of regulating the system in Eq. 1 to the predetermined physiological target value 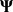, the quadratic cost function is defined as follows.
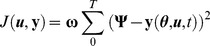
(12)


Where 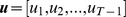
is the optimization vector consisting of all the control inputs for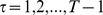. When more than one clinical target is optimized i.e. WBC as well as MCV, the errors are added and weighted appropriately with 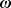 for the two targets. The constrained finite time optimal control problem can be formulated as follows,
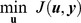
(13)subject to
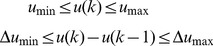
(14)where 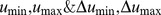 are constraints on range and slew rate of the 6-MP dose respectively. The restriction on slew rate is included to avoid extreme changes in dosage that will trigger drastic or negligible changes in associated cell population.

## Results

### 3.1 Parameter Identification for Average Patient Model

#### 3.1.1. 6-MP Model

**Figure 4:**
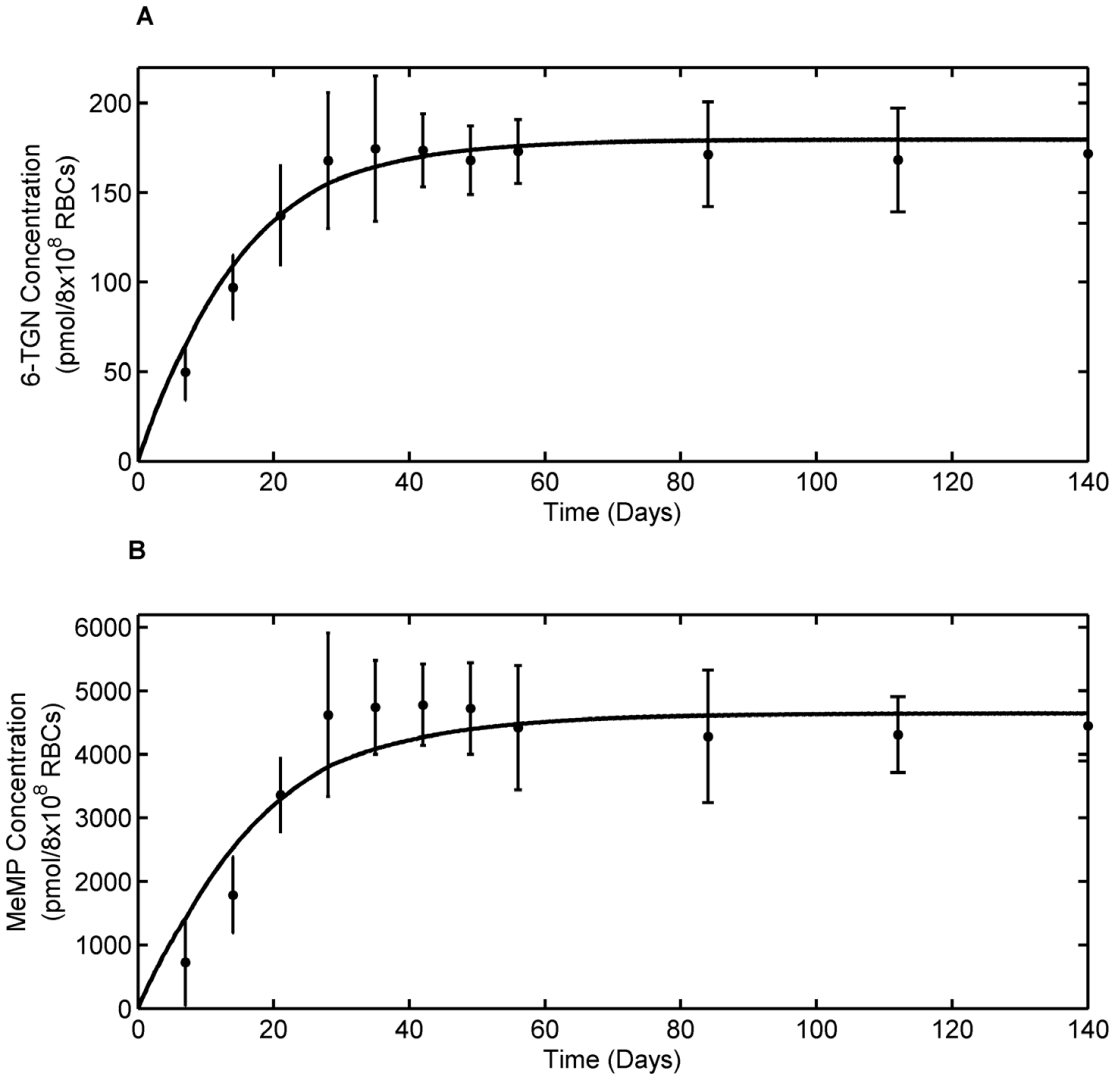
Average patient 6-MP model fit to literature data. A. Model fit to RBC 6-TGN concentration data. B. Model fit to RBC MeMP concentration data. Solid dots represent data points and curves represent 6-MP model (Eqn. 2) fit. Error bars represent standard error.

The data for estimating the parameters in the average patient model was collected from patients undergoing 6-MP treatment (“per protocol” group; n = 8) [50]. The dose schedule was assumed for a 70kg patient. Dose schedules for patients who encountered severe toxicity were altered but information pertaining to the modification was not given. Moreover, 6-TGN concentration data do not reflect major variation in the metabolite levels. Hence, we assume stable dose over the treatment period. Feasible range of parameters were chosen as follows: The range for *k_e_* was obtained from reported mean 6-MP plasma half-life of 1.2 hrs (range: 0.4–3.3 hrs) [51]. The range for 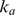
was estimated from lumping absorption rate and bioavailability reported in [34]. Conversion rates 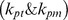 and M-M constants
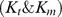
were estimated from 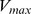
and 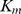
values given in [36], [52]. The elimination rates for metabolites 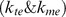
were estimated using elimination data from rat [53]. 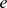
and 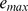
were fixed at 13 (median) and 26 (maximum) as assessed in [50], [54]. The average patient model fit to clinical data is shown in Fig. 4 and the estimated parameters are given in Table 1. The estimated parameters are observed to be in the ranges reported in the literature. It can be seen from the figure that the model fits the 6-TGN and MeMP data very well. Given the inter-assay coefficients of variation for 6-TGN and MeMP of 18% and 22%, respectively, the model predictions are extremely good.

#### 3.1.2. Leukopoiesis Model

The data for estimating the parameters in the leukopoiesis model in eqn. 4 was obtained from the same study as that of 6-MP metabolism data [55]. The leukocytes count for individual patients (n = 8) was used to calculate the average leukocyte count. The initial bounds for parameter estimation were chosen based on the physiological observation. 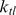 was chosen based on the mean transit time required for differentiation and maturation. 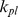 is slightly more than or equal to *k_tl_*, taking into account the death rate in the proliferation fraction of the cells. The range for 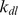 was chosen based on the half-life of leukocytes [56]. 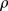 accounts for the variation in the proliferation rate when the circulating leukocytes deviate from the baseline, so steady state levels of leukocytes was chosen as a range. The steepness parameter 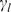 was assumed to be positive to impart negative feedback. The range for 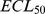
was chosen closer to observed concentration of 6-TGN in leukemia patients. A conversion factor of 15.5 kg/liter of blood was used to convert the cells per kg to cells per liter blood in the equation for leukocytes.

**Figure 5:**
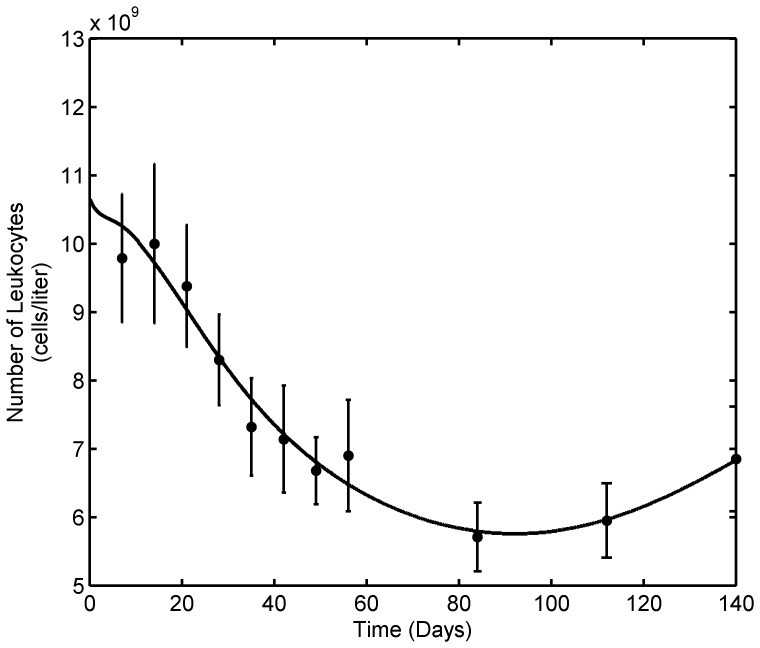
Leukopoiesis model fit to average patient data. Solid dots represent average patient data and curve represent leukocyte model (Eqn. 4) fit. The model mimics the clinical observation during 6-MP treatment; Depletion of leukocytes following 6-MP dosing has been countered by the body's feed-back mechanism. Error bars represent standard error.

The estimated parameter values of the leukopoiesis model for average patient are listed in Table 2 and the model fit to the average patient data is shown in Fig. 5. At steady state, 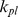 is estimated to be 0.157 day^−1^, which is slightly higher than 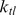 value of 0.1207 day^−1^ as expected. The difference accounts for the random death of cells in the proliferating progenitor compartment which is not explicitly considered in the model. The estimated value of 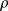 is in the vicinity of steady state leukocyte values. The value of *k_tl_* results in a slightly higher mean residence time for bone marrow population but it is anticipated given the fact that the inter-compartmental transfer rates are assumed constant in order to minimize the number of parameters. *ECL_50_* value of 84 pmol/8×10^8^ RBCs is within the expected range considering the 6-TGN concentration of 100–250 pmol/8×10^8^ RBCs observed in many human studies.

#### 3.1.3. MCV Model

**Figure 6:**
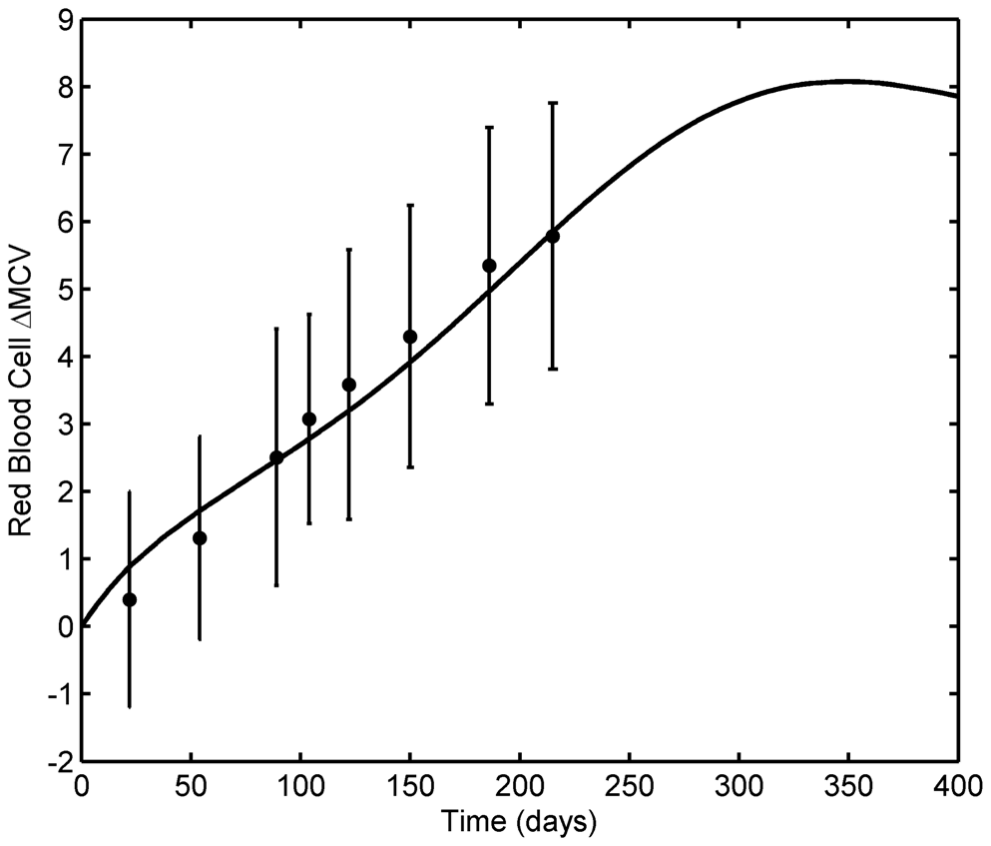
MCV model fit to average patient MCV data. Solid dots represent the data from literature and the solid line shows the model (Eqn. 7) fit with 6-TGN concentration of 158 pmol/8×10^8^ RBCs. The model fits the data well; it reaches the steady state and stays at ΔMCV of ∼8 fL, which is typically observed during 6-MP treatment at Riley Hospital for Children. Error bars represent standard error.

The experimental data for average patient MCV model (eqn. 7) parameter estimation was collected from published works [23]. Initial conditions were chosen based on the physiological values for bone marrow erythroid population, RBCs and MCV [40]. Bounds for parameter estimation was chosen based on the biology of the erythropoiesis like bone marrow residence time, half-life of RBCs and our previous work [25]. Fig. 6 shows the MCV model fit to the average patient data. The estimated parameters are listed in Table 3. The steady state value of *k_pr_* is slightly higher than the transfer rate as observed in the leukopoiesis model. The estimated value of feedback parameter 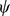 is in the range of steady state RBC count which is essential to impart proliferation amplification should the number of RBCs drop. The steepness parameter 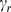 is much less than 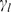 which shows that the feedback process is not as strong as that for leukocyte. This is in expected line given the fact that the turnover rate of RBCs is not as high as leukocytes. The values for *k_dr_* and 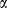
are close to physiological and literature values [23].

### 3.2. Model Reduction for Individualization

Variables for GSA and model reduction are the clinically measurable outputs, viz. 6-TGN concentration (*x_t_*), leukocyte number (*L*), and RBC MCV (*M*) from the three models respectively. The probability of the actual error 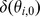, involved in fixing any parameter 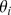 at 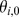, remaining below a certain level was assumed to be more than or equal to 0.95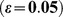. Since the sensitivity indices vary over the treatment period, we assumed six representative time points across the treatment period and cumulative error was calculated using Eqn. 12. 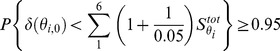
(15)


Any parameter, for which the error involved is less than 5% of the error associated with the most sensitive parameter, will be regarded as redundant and hence fixed at the average patient value for all individual patients. The sensitive parameters considered for individualization of each model are listed (in bold letters) with corresponding errors in Table 4. For 6-TGN model, the important parameters are the ones closely related to production and elimination of the active metabolite of our interest as observed in population studies [34]. This also points out that the conversion rate is the rate limiting step during 6-TGN production. Feedback mechanism and age related death are important regulation steps during hematopoiesis and helps to maintain the balance between the resources and body requirements [57]. These parameters together with death rate due to drug have naturally come out to be the sensitive ones in WBC and MCV models.

**Table 4:** List of parameters identified for individualization through GSA (in bold letters) together with other fixed parameters.

6-MP Model (Variable for GSA: *x_t_*)		
Parameters	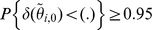	% error(actual/maximum)
***k_pt_***	**84.61**	**100**
***k_te_***	**46.36**	**54.8**
*K_t_*	0.0003	3.5×10^−4^
*k_e_*	0.0002	2.4×10^−4^
*k_a_*	4.0×10^−5^	4.7×10^−5^
*k_pm_*	2.1×10^−9^	2.5×10^−9^
*K_m_*	2.1×10^−9^	2.5×10^−9^
***k_me_***	0	0
**Leukopoiesis Model (Variable for GSA: *L*)**		
***k_tl_***	**65.54**	**100**
** 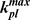 **	**49.12**	**74.9**
***k_dl_***	**39.02**	**59.5**
** 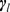 **	**22.61**	**34.5**
***EL_max_***	**17.60**	**26.9**
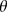	2.18	3.32
***ECL_50_***	**0.25**	0.38
**MCV Model (Variable for GSA: *M*)**		
***k_tr_***	**76.48**	**100**
** 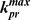 **	**50.09**	**65.5**
***k_dr_***	**20.71**	**27.1**
** 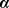 **	**11.00**	**14.4**
***ER_max_***	**10.49**	**13.7**
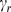	2.48	3.24
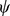	0.71	0.93
*ECR_50_*	0.0034	0.004

### 3.3. Model Individualization

**Figure 7:**
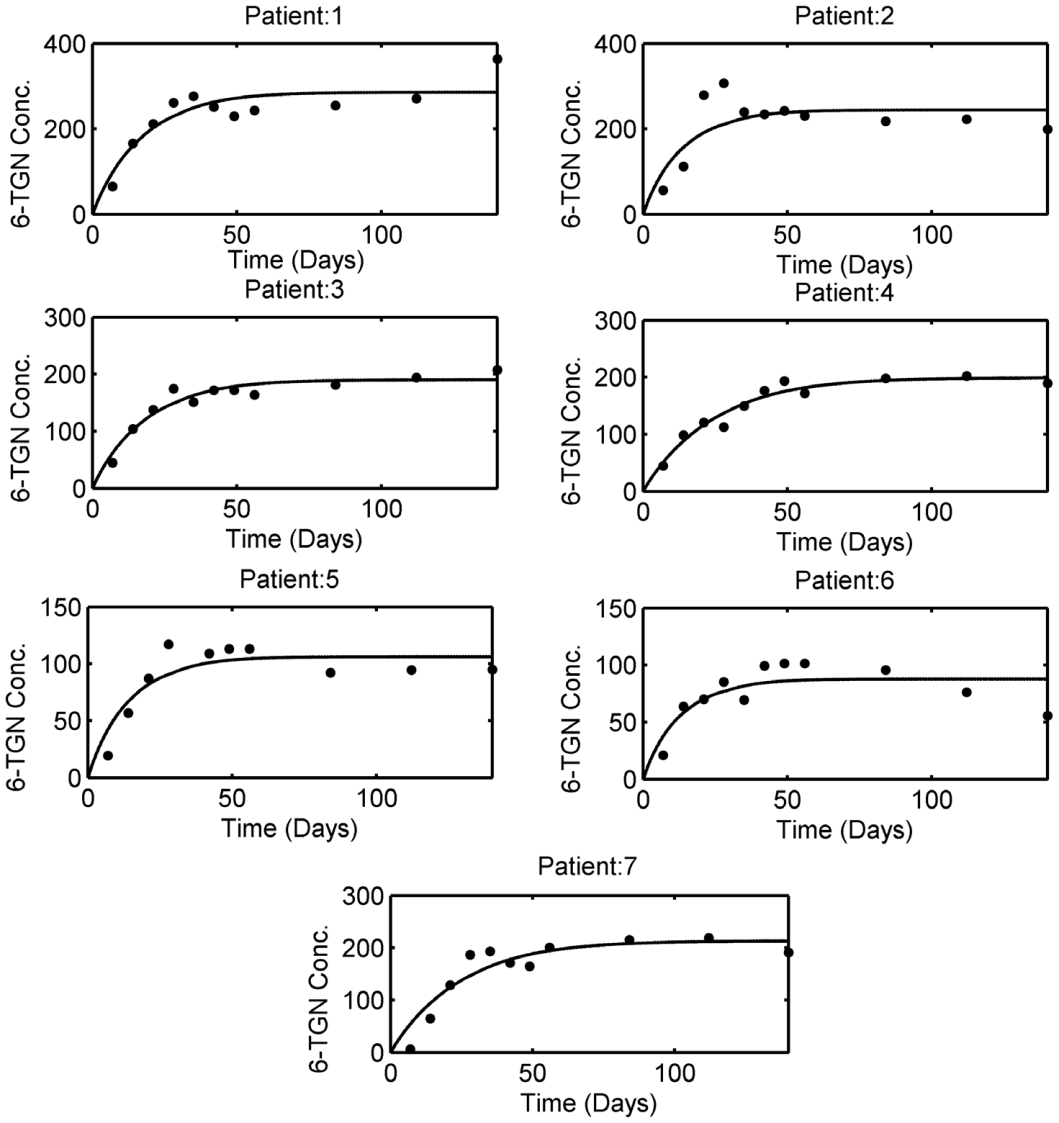
6-MP model fit to individual patient data obtained from literature. Solid dots indicate the individual patient 6-TGN concentration and the solid line represents the model fit. Patient # 8 was omitted from analysis as it is observed that the dosing was discontinued or substantially lowered. The estimated patient-specific parameters are provided in Table 5.

**Figure 8:**
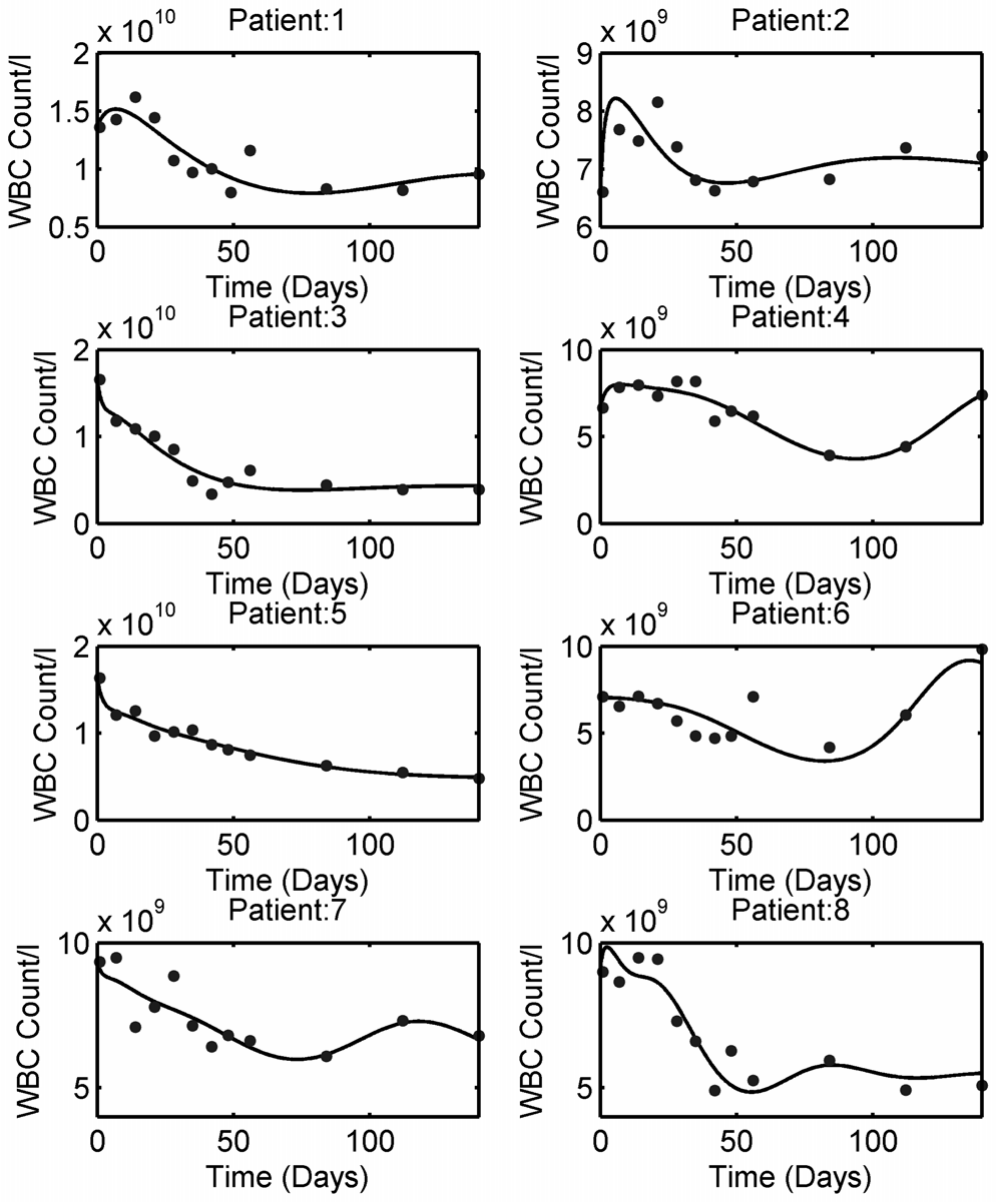
Leukopoiesis model fit to individual patient data obtained from literature. Solid dots indicate the individual patient WBC count and solid lines represent the model fit. The estimated patient-specific parameters are provided in Table 5. The model mimics diverse behavior observed during 6-MP treatment.

The 6-TGN concentration and leukocyte count data for individual patients were obtained from [55]. The model fitting to the individual patient data is shown in Fig. 7 and the estimated individual parameters are listed in Table 5. In light of the reported inter-assay coefficient of variation for 6-TGN (18%), the model fits the data reasonably well. For leukocyte data, individual patients were not identified in the plots for 6-TGN concentration and leukocyte count in the original data source. Hence, average patient 6-MP model parameters were used to simulate the leukopoiesis model. The model fitting to the individual patient leukocyte data is shown in Fig. 8 and the estimated individual parameters are listed in Table 5. From the figure, it is evident that each patient responds to the treatment differently. In some patients, the feedback mechanism is strong enough to return the leukocytes towards the desired level whereas in others, the feedback is weak which is a common phenomenon in leukopoiesis [42].

**Table 5:** Patient-specific parameters estimated for 6-MP, leukopoiesis and MCV models.

6-MP Model		
Pat. No.	*k_pt_*	*k_te_*
1	38.4	0.0646
2	41.4	0.0815
3	23.9	0.0604
4	18.76	0.0453
5	17.4	0.0788
6	15.83	0.0867
7	20.04	0.0452
Mean	25.1	0.0661
SD	10.45	0.017
Min	15.83	0.0452
Max	41.4	0.0867

**Figure 9:**
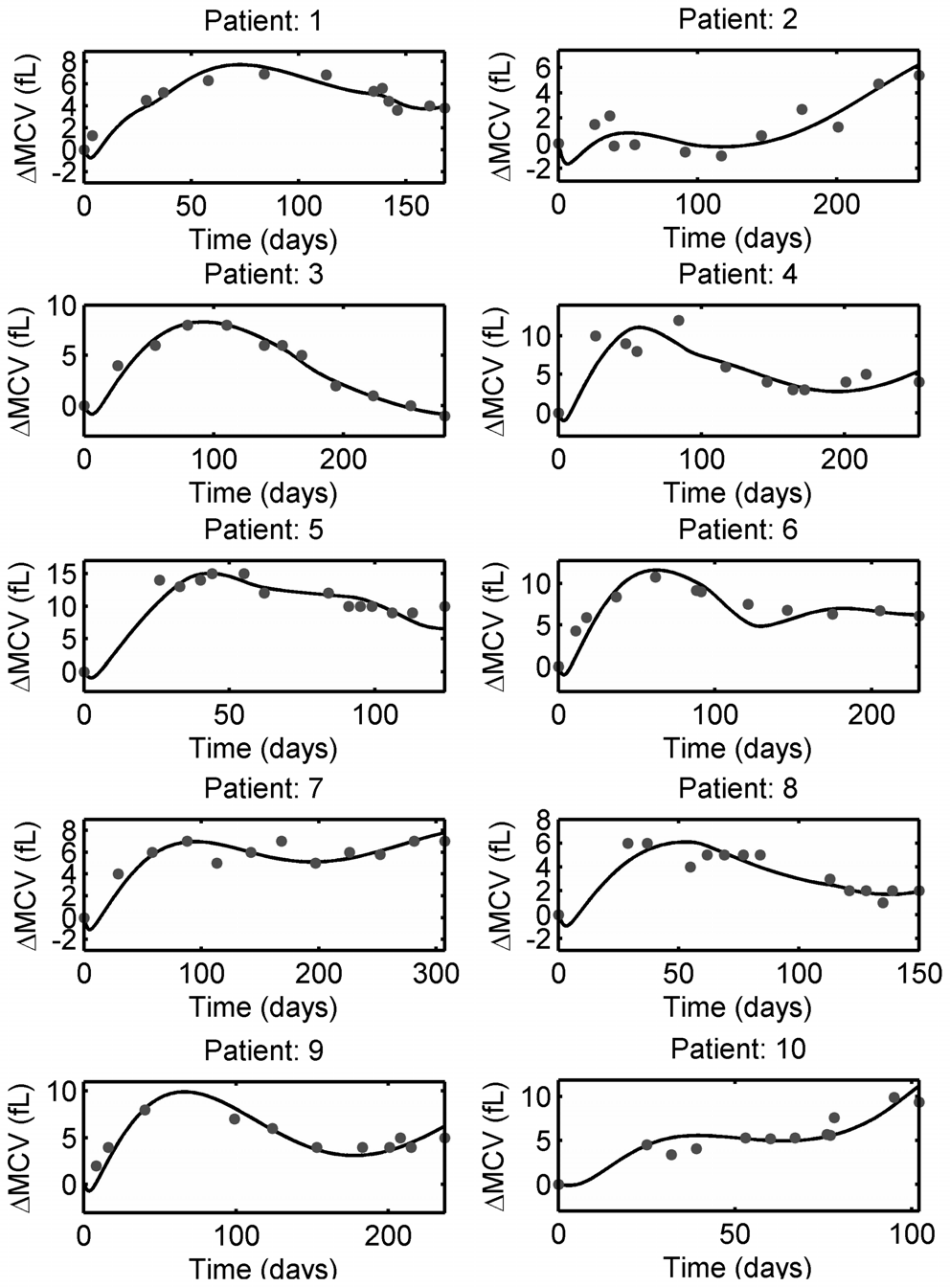
MCV model fit to individual patient data obtained from Riley Hospital for Children. Solid dots indicate ΔMCV of individual patient undergoing 6-MP treatment and solid lines represent the model fit. The estimated patient-specific parameters are provided in Table 5.

Individual MCV measurements from pediatric ALL patients were obtained from Riley Hospital for Children in Indianapolis. The data was de-identified and collected according to approved IRB protocol (0505002519). Patients were undergoing MT with 6-MP and MTX. Patients were given a standard initial 6-MP dose of 75 mg/m^2^ and subsequent doses were adjusted in accordance with observed leukocyte count. Hence, the dosing pattern and dosage varied substantially among and within patients. 6-TGN levels were not measured in these patients, so average parameters were used in 6-MP model. The model fitting to individual patient data are shown in Fig. 9 and the estimated parameters are listed in Table 5.

#### 3.3.1. Predicting Response through Virtual Patient Simulation

**Figure 10:**
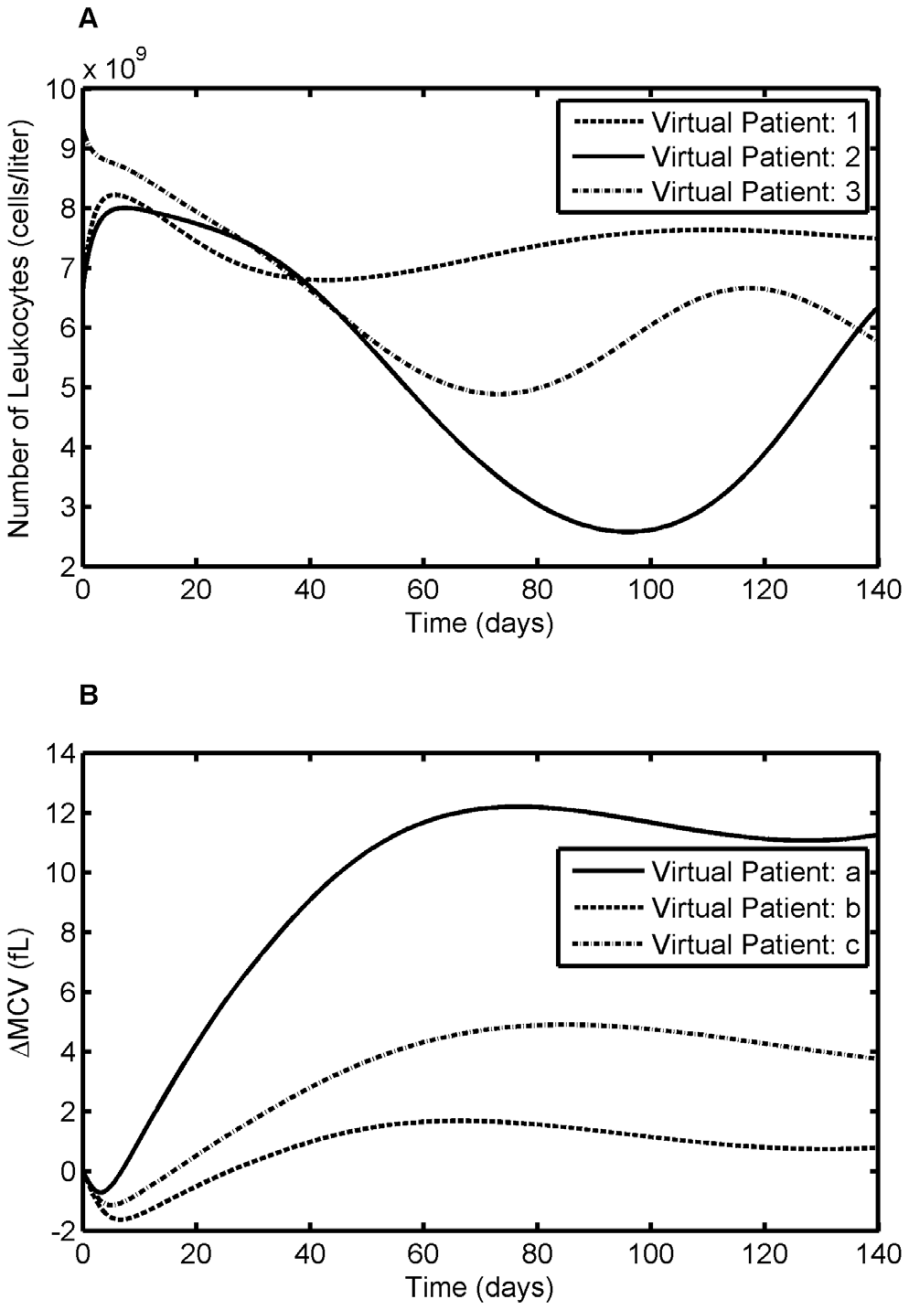
Virtual patient simulation for leukocyte and MCV model. Data for 6-MP model and leukocyte model are assumed to have originated from the same patient. The resultant estimated parameters for three representative patients are used to simulate the virtual patient response. It is apparent from the figure that different patients achieved different levels of response for the same dose, thereby achieving different treatment outcome. A. Leukocyte model, B. MCV model.

In order to appraise the variations in response to the treatment, we have created virtual patients by assuming a correspondence across the parameter sets of the three models, i.e. all possibilities of parameter set combinations across three models. This has produced 56 virtual patients for leukocyte response and 70 virtual patients for MCV response. Figures 10A & 10B show three representative patient responses for leukocytes and RBCs, respectively. It is apparent from these figures that, for the same pattern of drug dosing, individual patients varied widely in their response. The comparison between the published results and model predicted values for the three models are summarized in Table 6. The results are statistically significant using t-test (for mean comparison) and F-test (for variance comparison) and thus show that the model prediction and clinical results are the same. The model predicted standard deviation (SD) for ΔMCV is significantly higher than the literature values as there were many dose adjustments in our study; whereas Decaux et al. study was conducted at stable dose.

**Table 6:** Comparison between model-predicted values (mean ± SD) and published clinical results for model response variables.

Variables	Published Values [23]	Model Prediction	p-value (mean)	p-value (variance)
6- TGN	176.0±97.63 (n = 37)	172.18±71.0 (n = 7)	0.9034	0.2175
ΔMCV	5.8±3.58 (n = 37)	5.95±5.17 (n = 70)	0.8606	0.009
Leukocytes	6.43±2.61 (n = 20)	5.93±2.33 (n = 56)	0.4526	0.2511

**Figure 11:**
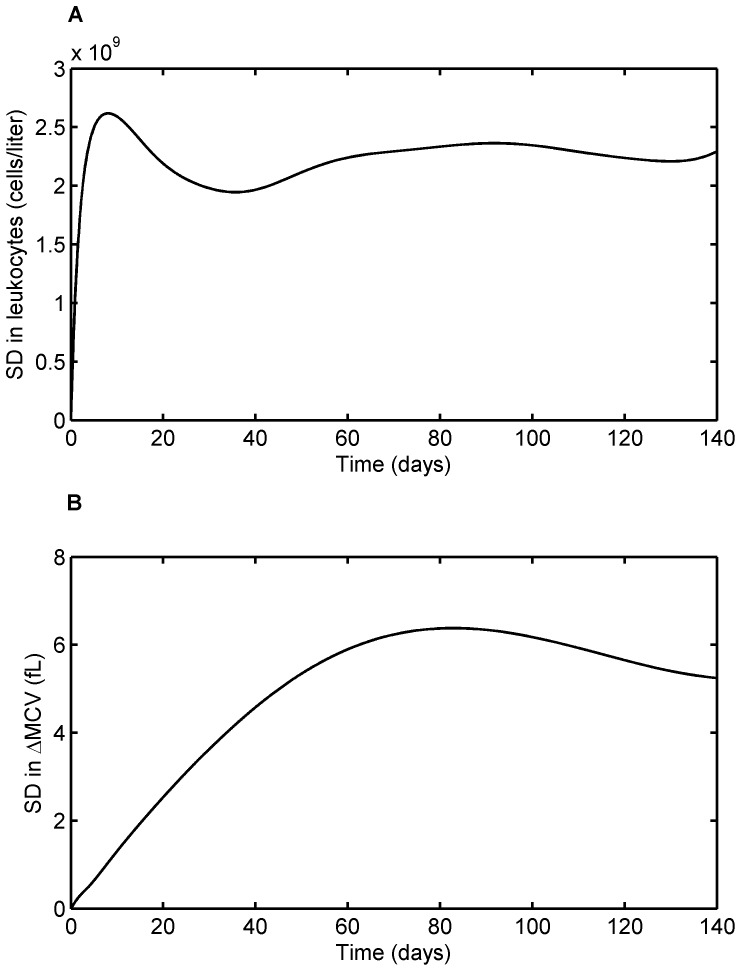
Model-predicted standard deviation in the treatment response over treatment period. A. Standard deviation for leukocytes response as a function of time, B. Standard deviation for ΔMCV. Variability explained by the models is in line with the pattern observed during 6-MP treatment.

Fig. 11 shows the profiles of standard deviation in leukocyte and MCV responses. The variations predicted by the models are physiologically anticipated. For leukocytes, the variation spikes at the start of the therapy as there are huge variation in response to the therapy. As the treatment progresses, the feedback mechanism gets underway and brings the leukocyte population back to the base line which is relatively constant for all the patients. This helps to subside the variation at the later stages of the treatment. In contrast, for ΔMCV, the variation at the onset of the treatment is small compared to that of the later stage. This is expected since the treatment response is based on the MCV change after four months of continuous therapy.

In order to validate the model's ability to predict future response, we estimated parameters for individual patients with 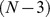 original data points. With these parameters, the remaining data points were predicted and the SSEs are calculated for full and 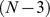 data points. For all patients, the differences in SSEs are less than 2% for 6-MP model, and less than 5% for leukocyte and MCV models.

### 3.4. Dose Optimization

**Figure 12:**
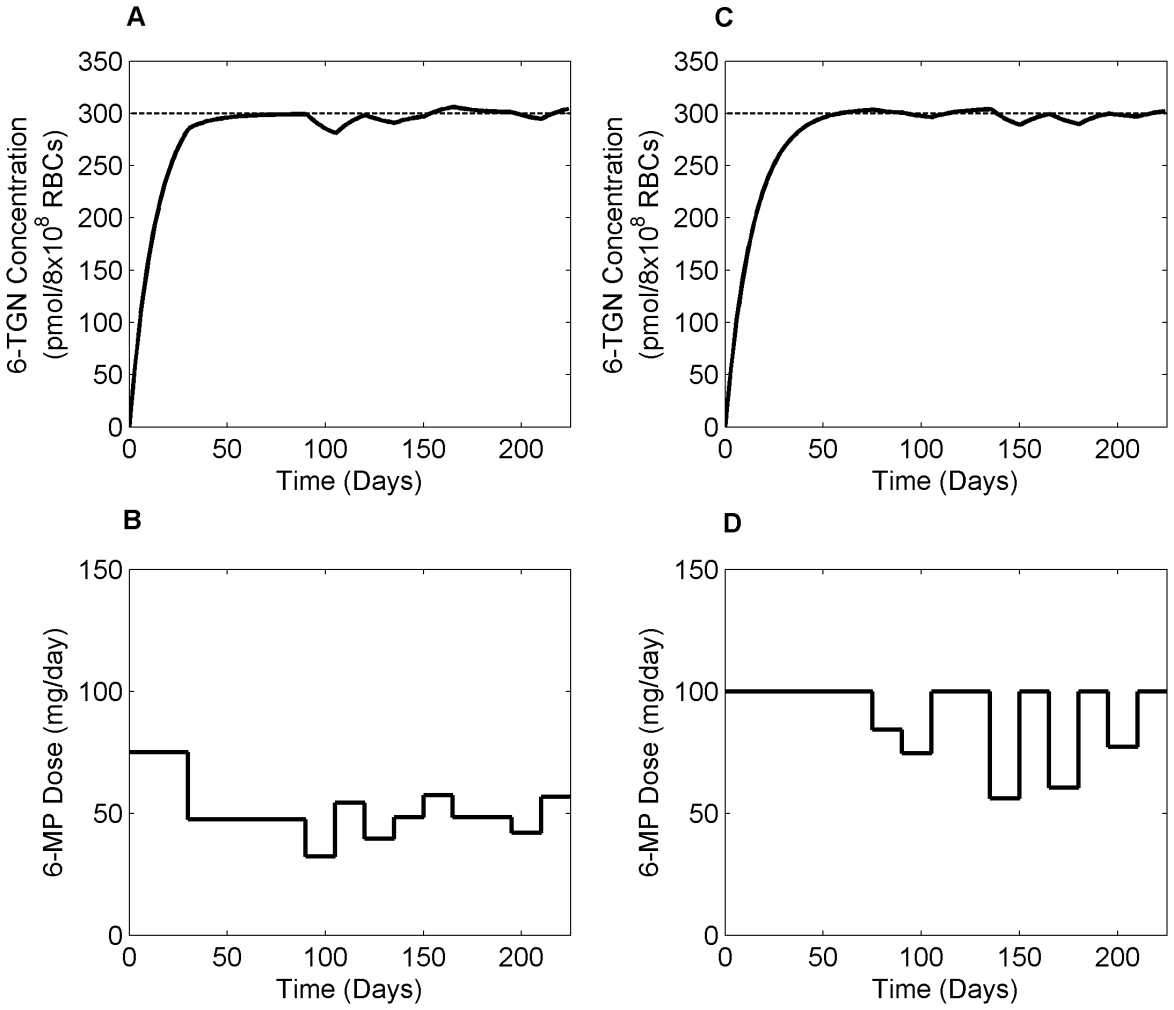
Optimal dosing based on 6-TGN concentration as therapeutic target. A & B. Optimal 6-TGN concentration and optimal 6-MP regimen respectively, for a patient with low TPMT enzyme activity. C & D. Optimal profiles for a patient with high TPMT enzyme activity. Patient with low TPMT activity required significantly lower dose compared to the standard treatment and vice versa. Dashed line shows the therapeutic target level.

**Figure 13:**
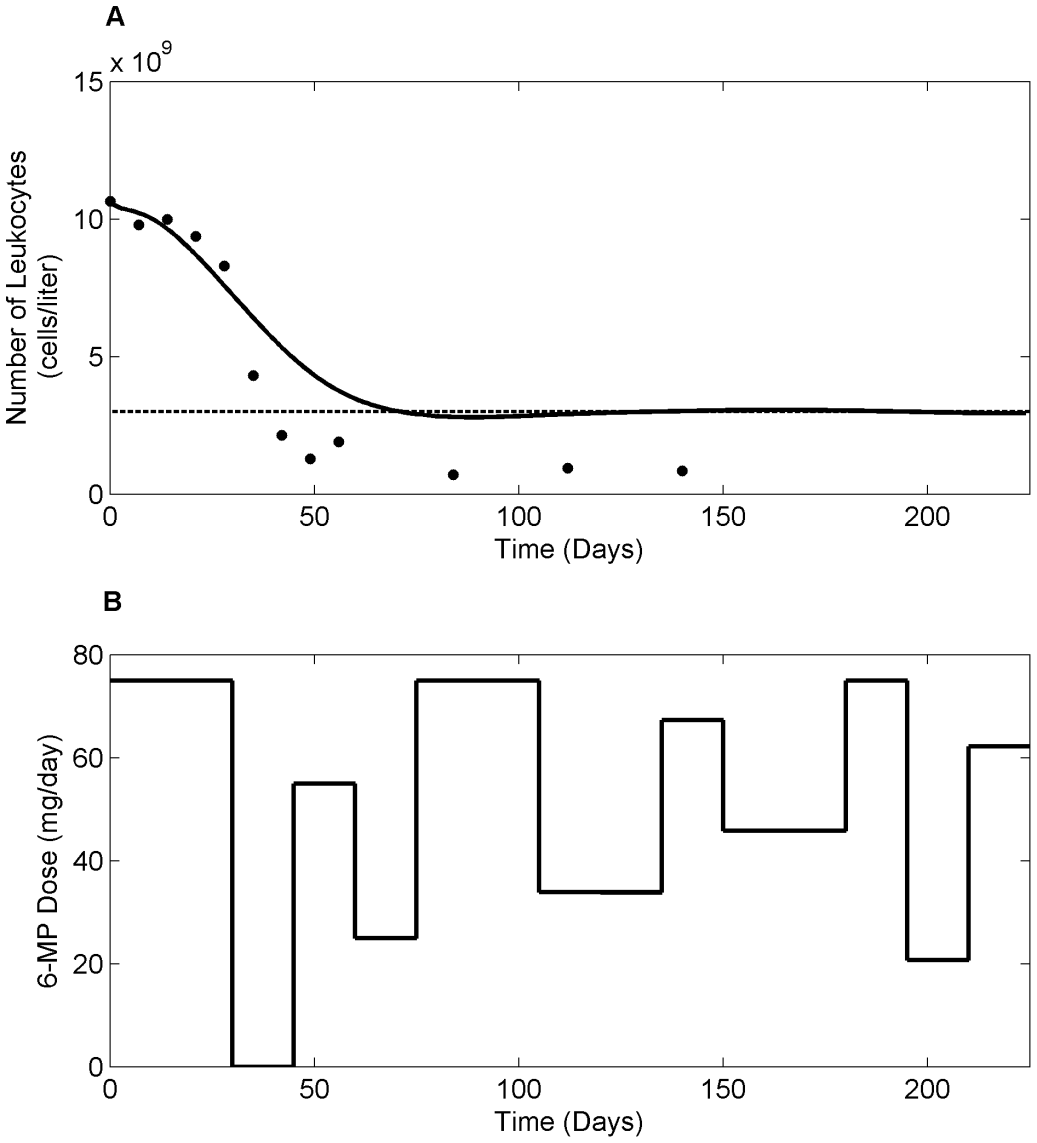
Optimal dosing based on leukocyte count as target. A. Evolution of leukocyte count in response to optimum 6-MP dosing. Dashed line represents critical leukocyte level and solid dots represent clinical data for an average patient. B. Optimum 6-MP dosing profile predicted by NMPC. The standard daily 6-MP dosing is 75 mg/day.

**Figure 14:**
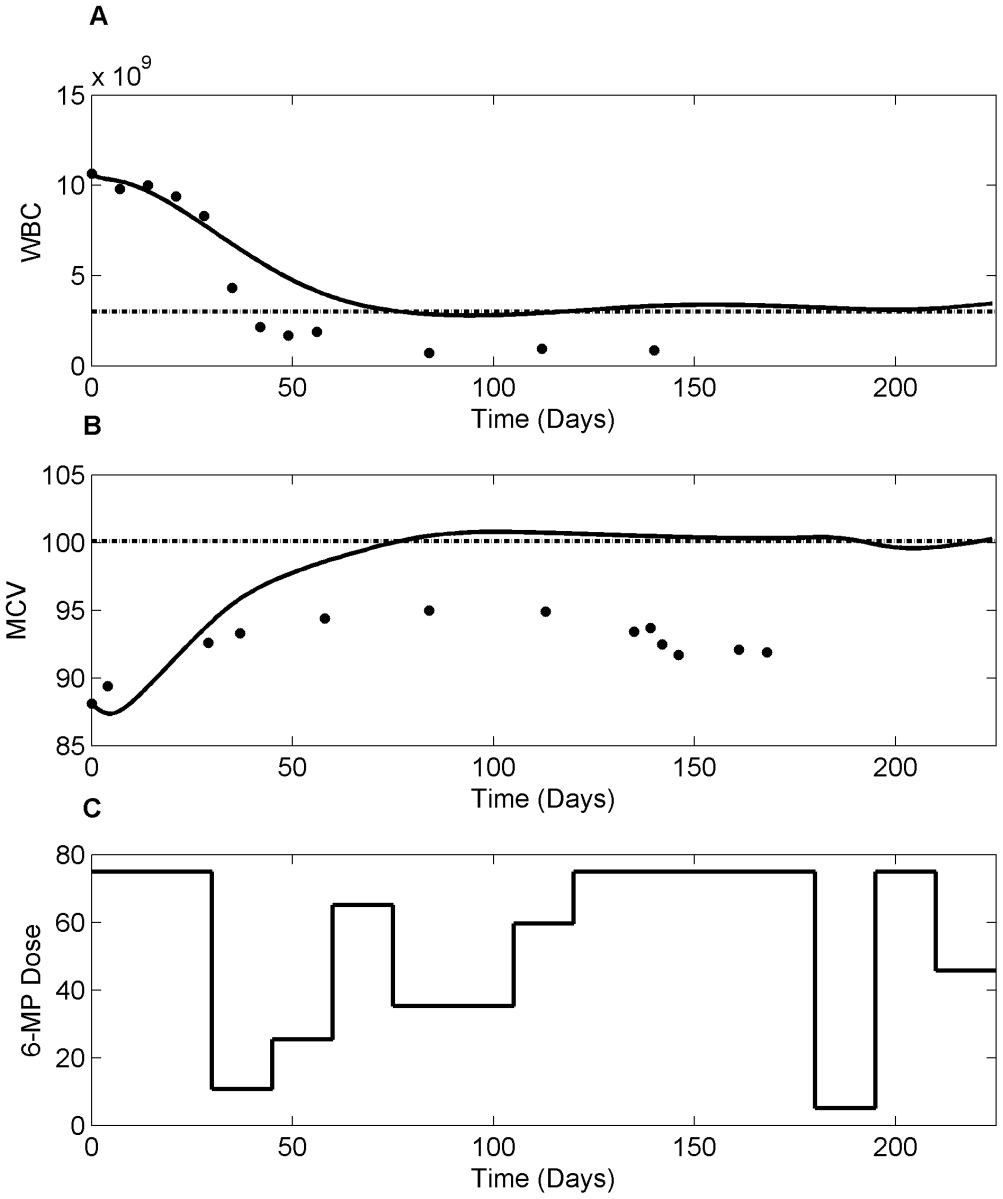
Optimal dosing based on leukocyte count and MCV as target. A. Evolution of leukocyte count in response to optimum 6-MP dosing. B. Evolution of ΔMCV response with optimum 6-MP dose. Dashed lines represent critical leukocyte and target MCV levels and solid dots represent clinical data for an average patient. C. Optimum 6-MP dosing profile predicted by NMPC. The standard daily 6-MP dosing is 75 mg/day.

NMPC control strategy was applied to clinical scenarios using three different therapeutic targets: i) 6-TGN, ii) leukocyte count, iii) both leukocyte count and ΔMCV. All dosing calculations are based on 15 days sampling horizon with 75 days treatment window. Clinical studies propose a therapeutic window of 235–400 pmol/8×10^8^ RBCs for 6-TGN concentration for effective management of both efficacy and toxicity [20], [58]. Hence, we optimized 6-MP input with a target 6-TGN concentration of 300 pmol/8×10^8^ RBCs. Fig. 12 shows the optimal 6-MP input together with resultant 6-TGN concentration for low and high TPMT activity patients. Dose inputs for different patients suggest that dosage varies mainly as a function of the 6-TGN conversion rate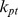. From the figure, low TPMT patient required 31.82% less dose compared to the standard dose whereas, high TPMT patient needed 20.25% higher. Although 6-TGN concentration is not an ideal target, as there are further variability in pharmacodynamics, it proves useful in certain clinical conditions where efficacy measures are categorical and highly subjective. Fig. 13 shows the optimal dosage for current clinical practice where the dosing decisions are primarily driven by leukocyte count. Currently, patients are ‘titrated’ to a target leukocyte level of 3×10^9^ cells/liter using a trial-and-error approach. However, with the help of NMPC, the oncoming leukopenia is predicted and dosing adjustments are made in a timely manner so that the leukocyte count remained close to the critical level for fighting infection. Fig. 14 shows the optimal 6-MP profile with simultaneous optimization of both therapeutic targets, i.e. maximizing efficacy without causing severe toxicity. The significance of these dose optimization should be viewed from the maximization of therapeutic benefits rather than the reduction of drug input as the cost of drug is only a fraction of the overall healthcare spending. Interestingly, cumulative optimal dosage for some of the patients are equivalent to their actual cumulative clinical dosage, but timely reduction or suspension of treatment predicted by NMPC has proven vital for forestalling life-threatening toxicity or compromising efficacy.

## Discussion

We have presented here a modeling and individualized dosing approach to predict the treatment outcome and optimize the dosage during chemotherapy of childhood ALL. It should become transparent from this paper and other similar efforts [59], [60] that mathematical models could in fact be an eminent substitute for empirical treatment adjustments. Mathematical models were developed and validated for 6-MP metabolism, MCV dynamics (a surrogate marker for treatment efficacy) and leukopenia (a dose-limiting side-effect during treatment). The models were fitted to real patient data obtained from clinical settings. It is evident from the figures 8–10 that the models fit the data well with reduced parameter space. It is important to note that the data needed for this study did not come from the same source so we had to assume that the 6-TGN concentration was that of an average patient in leukopoiesis and MCV models. Some of the variations observed in the patient-specific parameters of leukopoiesis and MCV models may have arisen from the fact that the patients have performed significantly different from the average patient's 6-TGN concentration.

The implementation of the proposed approach in clinical practice can be envisioned as follows: When the new patient completes the induction treatment and is ready to start the 6-MP based consolidation and maintenance therapy, TPMT enzyme activity has to be measured. This actual TPMT enzyme activity will replace the estimate *e* in the 6-MP model and dictate the initial 6-MP dosing based on therapeutic window based NMPC. As the metabolite measurements and CBC become available, parameters can be progressively estimated from the most sensitive to the least sensitive in the reduced parameter group and the models are adapted to the new patient. Once a patient-specific model is obtained, future course of action can be predicted and optimized using NMPC.

It is a known fact that the global sensitivity indices for various parameters are not identical at different time points. Given the fact that only a few sensitive parameters are estimated to individualize the model, future experiments for new patients have to be designed carefully to estimate these parameters with precision. In this line, dynamic model-based design of experiment strategies can be exploited to estimate the optimum measurement points and other design variables. Efforts are in progress to determine the optimal experimental points for each of these models and compare the parameter precision and prediction capabilities of the models with a reduced data set.

It has been the aim of this work to address issues concerning the survival and well-being of children afflicted by ALL. The methodology, however, has import for treatment of other cancers or diseases as well where treatment involves the same or similar drugs. For instance, 6-MP is the major drug in treating autoimmune diseases and inflammatory bowel disease (IBD) whose incidence rate far exceeds that of ALL. Similar issues to that of ALL have been reported in IBD treatment with 6-MP. Hence the proposed techniques have the potential to be extended to a broader class of diseases and medical conditions. Admittedly, besides the technical solutions described here, there are other challenges to individualized treatment such as physiological, logistical, economical and societal in nature. When addressed together, it has the potential to reduce healthcare cost as well as improve the quality-of-life among patients by effectively tuning the therapy for each patient based upon their own response instead of the statistics of prior trials.
